# The Insensitive Ruins It All: Compositional and Compilational Influences of Social Sensitivity on Collective Intelligence in Groups

**DOI:** 10.3389/fpsyg.2016.00676

**Published:** 2016-05-09

**Authors:** Nicoleta Meslec, Ishani Aggarwal, Petru L. Curseu

**Affiliations:** ^1^Department of Organisation Studies, Tilburg UniversityTilburg, Netherlands; ^2^Brazilian School of Public and Business Administration, Getulio Vargas FoundationRio de Janeiro, Brazil; ^3^Department of Psychology, Babes-Bolyai UniversityCluj-Napoca, Romania; ^4^Department of Organisation, Open University of the NetherlandsHeerlen, Netherlands

**Keywords:** collective competencies, group composition, social sensitivity, group synergy, collective intelligence

## Abstract

A group's collective intelligence reflects its capacity to perform well across a variety of cognitive tasks and it transcends the individual intelligence of its members. Previous research shows that group members' social sensitivity is a potential antecedent of collective intelligence, yet it is still unclear whether individual or group-level indices are responsible for the positive association between social sensitivity and collective intelligence. In a comprehensive manner, we test the extent to which both compositional (lowest and highest individual score) and compilational aspects (emergent group level) of social sensitivity are associated with collective intelligence. This study has implications for research that explores groups as information processors, and for group design as it indicates how a group should be composed with respect to social sensitivity if the group is to reach high levels of collective intelligence. Our empirical results indicate that collectively intelligent groups are those in which the least socially sensitive group member has a rather high score on social sensitivity. Differently stated, (socially sensitive) group members cannot compensate for the lack of social sensitivity of the other group members.

## Introduction

Small social groups are information-processing units (Hinsz et al., 1997; Hinsz, 2015), extensively used in organizations to solve problems, make important decisions and generate innovations (Devine et al., 1999). In order to tackle these complex cognitive problems, groups combine, through social interactions, the cognitive resources (knowledge, skills, abilities; Kozlowski and Ilgen, 2006) of their members in collective, group-level structures and competencies. In other words, groups are cognitive systems with emergent cognitive competencies that transcend the cognitions of their individual members (Woolley et al., 2010; Curşeu et al., 2013).

An integrative framework that explains the effectiveness of these collective cognitive systems is the teams as cognitive technology framework (Wallace and Hinsz, 2010; Hinsz, 2015). This framework argues that teams are a form of human technology and the use of teams can be seen as a method for solving practical organizational problems, just like the use of any other technology (Hinsz, 2015). In line with this framework, a group's cognitive performance results from the transformation of the cognitive resources brought in the group by its individual members. The quality of interpersonal interactions is critical for the transformation of the cognitive resources within groups and for the emergence of group cognition (Curşeu et al., 2007). Therefore, team members need good interpersonal skills in order to be collectively successful in cognitive tasks.

Recent empirical studies have provided substantial support for a collective intelligence (CI) factor that describes a group's systematic capacity of performing effectively on a variety of cognitive tasks (Engel et al., 2014, 2015; Woolley et al., 2015 for a review; Woolley et al., 2010). The strongest predictor of collective intelligence seems to be the average social sensitivity of the group members (Woolley et al., 2010). Social sensitivity represents the ability of individuals to accurately attribute mental states to oneself or another person (Premack and Woodruff, 1978; Baron-Cohen et al., 2001). In other words, it represents the ability of group members to put themselves in the mind of other persons and “tune in” to their mental states (Baron-Cohen et al., 2001). Social sensitivity is responsible for the way in which we make sense of and predict behaviors of other people (Baron-Cohen et al., 2001). In groups, harmonious interpersonal interactions are facilitated by rewarding social exchanges and helping behaviors (Berkowitz, 1970; Homans, 1974). Social sensitivity is likely to facilitate these behaviors through group members' ability to perceive each other's mental states, and hence is a prerequisite for harmonious interpersonal interactions in groups.

Socially sensitive group members attend to the interpersonal dynamics in groups and create a positive interpersonal atmosphere that is ultimately conducive to task performance (Curşeu et al., 2015a). Therefore, collective intelligence, as a set of cognitive competencies that emerge from the task-directed interpersonal interactions in groups, depends on members' capacity to accurately perceive their social environment as demonstrated by the strong relationship between group members' social sensitivity and the collective intelligence of the group (Woolley et al., 2010). Further research is needed to understand the role of social sensitivity as a source of collective intelligence in a comprehensive manner; i.e., while it has been reported that the average social sensitivity of group members is associated with collective intelligence (Woolley et al., 2010), the role of other group aggregates (e.g., the minimum social sensitivity score or the maximum social sensitivity score) has not been yet explored.

In order to look at this relationship more intricately, we borrow from the approaches used in the team personality literature. In a comprehensive review on the effect of personality on team performance, LePine et al. (2011) argue that one needs to consider both team composition with respect to various personality traits of group members as well as the collective, emergent group personality. Group personality represents the behavioral regularities exerted by the group as a whole (Stewart, 2003; LePine et al., 2011). Because the estimated positive effect of average individual social sensitivity on CI could hide both individual (lowest or highest individual score effects) and group level effects (emergent group-level social sensitivity), one needs to distinguish between individual- and group- level social sensitivity. In addition to the theoretical implications, this distinction has important implications for group design as it clarifies how a group should be composed with respect to social sensitivity if the group is to be highly collective intelligent.

The main aim of our paper is to explore the association between different indices of social sensitivity in groups and CI. In line with team personality models (Barrick et al., 1998; LePine et al., 2011), we argue that the positive association between the highest/lowest individual social sensitivity score and CI is likely to reflect different convergence mechanisms explained by the compositional and compilational emergence logic described in the multi-level perspective on groups (Kozlowski and Chao, 2012; Kozlowski et al., 2013).

To summarize, our paper builds on the teams as cognitive technology framework (Hinsz, 2015) to argue that transformation processes embedded in interpersonal interactions are essential for the emergence of collective intelligence. We use the insights from group personality models (Prewett et al., 2009; LePine et al., 2011) to argue that social sensitivity is a key interpersonal attribute that should be considered at the individual as well as the group level of analysis. In order to disentangle the mechanisms that drive the association between social sensitivity and CI, we use the multi-level perspective on groups (Kozlowski and Chao, 2012; Kozlowski et al., 2013) to assess the compositional and compilational factors that could explain the positive association between social sensitivity and collective intelligence in groups. In order to distinguish between the compositional and compilational effects of social sensitivity on collective intelligence, we take into account both individual social sensitivity (and the way it is distributed within groups as the minimum and the maximum score) as well as collective social sensitivity (an attribute that describes the group as a whole).

## Compositional arguments

Literature to date (Barrick et al., 1998; Devine and Philips, 2001; LePine, 2003) distinguishes between conjunctive and disjunctive compositional aggregation models in teams. Conjunctive models assume that taking into account the lowest individual score has the highest predictive power for team-level effects. Disjunctive models, on the other hand, assume that the highest individual score is the best predictor for team outcomes. Previous research (Barrick et al., 1998; Devine and Philips, 2001; LePine, 2003) has associated these operational models with the type of task. According to Steiner (1972) unitary group tasks (where one single outcome needs to be generated per group and the task must be performed by the group as a whole) can be further classified as conjunctive, disjunctive, additive or discretionary. In a conjunctive task, the performance of the group is as high as the lowest performing individual. For example, the performance of a group of mountaineers who aim to reach the summit will always depend on the speed of the least capable member. In a disjunctive task, the performance of the group is as high as the highest performing individual in the group. For example, the performance of a group that aims to find a solution for a mathematical problem will depend on the problem-solving capabilities of its best member.

The parallel between the type of task (conjunctive or disjunctive) and the aggregation method (the minimum score and respectively the maximum score), however, does not fully clarify the way in which the lowest or the highest individual scores relate to group-level outcomes. We further draw on group diversity and social influence literatures (Moscovici, 1976; Tanford and Penrod, 1984; Nemeth, 1986; Wood et al., 1994), to distinguish between minority and majority influence as mechanisms that explain the positive association between the highest, and respectively the lowest, individual social sensitivity scores and CI in groups.

We argue that, if the highest individual social sensitivity score predicts CI, it means that minority influence processes were effective and explain the convergence of social sensitivity within groups. Minority dissent is a source of social influence in the group and is defined as the situation in which a minority in the group publicly opposes beliefs, attitudes or ideas held by the majority (Moscovici, 1976; McLeod et al., 1997). Minorities, through their opposing views, stimulate the reappraisal of the situation at hand and widen the attention of the group members to aspects that have not been previously considered (Nemeth, 1986; Nemeth and Kwan, 1987). As a consequence, minority dissent has been associated with increased group creativity (De Dreu and West, 2001; De Dreu, 2002), better problem-solving (Nemeth and Wachtler, 1983) and higher decision-making performance (Schulz-Hardt et al., 2006). Similarly, the most socially sensitive person in the group can confront the other members with a different and more accurate perspective on the socio-emotional dynamics of the group. As a result, the group as a whole is likely to broaden its attention to interpersonal dynamics and work with the emotional states experienced by the individual members. This comes in line with the cascading model of emotional intelligence indicating how emotional perception and understanding precedes emotional regulation (Joseph and Newman, 2010). Effective emotion regulation in groups improves the quality of interpersonal interactions and ultimately group performance (Curşeu et al., 2015a). To conclude, if the maximum individual score significantly and positively predicts CI, it would imply that minority influence processes were effective and they explain the association between social sensitivity and CI.

A significant and positive association between the lowest individual social sensitivity score and CI means that majority influence explains the convergence of social sensitivity within groups. Majority influence is a second source of social influence in groups and it reflects a situation in which attitudes, ideas and opinions held by the majority of the group will ultimately be shared by the group as a whole (Nemeth, 1986). Majorities have been found to stimulate convergence due to the implicit assumption held by the group members that the majority must be correct and due to the fear of disapproval associated with dissent (Nemeth, 1986). If the majority of the group members share high social sensitivity (indicated by a rather high score of the least sensitive group member), this attribute will ultimately converge, become manifested in interpersonal interactions and influence the group as a whole. In other words, groups need a particular threshold for social sensitivity in order to allow its expression in interpersonal interactions. If the lowest social sensitivity score significantly and positively predicts CI, it would imply that majority influence processes, leading to group-level convergence of social sensitivity, explain the association between social sensitivity and CI.

Based on this compositional logic and drawing on minority and majority influences we explore the extent to which the minimum level of social sensitivity (the group needs to meet a threshold of social sensitivity) as well as the maximum score within group (a highly sensitive person is enough) will be associated to collective intelligence in groups. Differentiating between the two scores allows us to identify the most likely social influence process that explains the positive association between social sensitivity and CI. This exploratory approach comes in line with similar studies looking at configurations of individual-based variables within groups such as personality (Barrick et al., 1998) or cognitive ability (Devine and Philips, 2001).

## Compilational arguments

Beyond the variability of individual social sensitivity within groups, this attribute can also describe groups as collectives. Collective induction processes help groups to combine the individual traits and competencies of their members in collective, group-level phenomena (Curşeu et al., 2015b). We build on the concept of collective personality (Stewart, 2003; LePine et al., 2011) to argue that groups may vary in their level of social sensitivity, and tasks used to evaluate individual social sensitivity can be extrapolated to evaluate between-group variability in social sensitivity. Collective personality is an emergent group-level construct that refers to the behavioral regularities of the group as a whole (Stewart, 2003; LePine et al., 2011). Collective personality, as opposed to individual personality, emerges from interpersonal interactions unfolded in the group (Kozlowski and Klein, 2000) and can be influenced by individual personalities but also other factors such as leadership (Hofmann and Jones, 2005) and interpersonal interactions in groups.

Similar arguments are used in the group synergy literature, in which collective performance is compared to average, or best, individual performance in order to evaluate the effectiveness of collective induction processes (Curşeu et al., 2015b). Following the group synergy procedures (where the same task is performed first by individuals and then by groups), we intend to evaluate collective social sensitivity by asking groups, as collectives, to perform the same (social sensitivity) task performed by their individual members. We expect that group's social sensitivity, as a collective trait is positively related to collective intelligence. A group that is capable, as a whole, to correctly identify other people's mental states and “tune in” accordingly, can use this ability as a self-regulation mechanism to enforce coordination processes, adjust to varying task demands and create a positive group atmosphere. Group social sensitivity, hence, is expected to be conducive for high levels of performance across multiple tasks, and ultimately to positively predict CI. A similar concept, collective emotional intelligence has been found to be positively associated with group effectiveness (Jordan and Troth, 2004; Curşeu et al., 2015a). Collective emotional intelligence is a group-level competence that reflects the ability of the group to develop a set of norms that encourage expression, awareness and regulation of affective dynamics within the group (Druskat and Wolff, 2001; Curşeu et al., 2015a).

To summarize, in line with previous research we expect that all three indicators: minimum, maximum and collective social sensitivity have a positive association with collective intelligence. We argue, however, that the association between these three indicators and collective intelligence is likely to be explained by different mechanisms. The main aim of this paper is to estimate the predictive power of these three variables simultaneously in order to comprehensively understand the association between social sensitivity and collective intelligence.

## Methods

### Sample and procedure

The sample consisted of 154 participants (78 women) organized in 30 student groups. Group size varied from 3 to 6 members (with a mean = 4.97, standard deviation = 0.60 and median = 5). Groups worked together for a period of 3 months in order to complete a research assignment for an organizational studies course at a Dutch university. Groups were formed at the beginning of the semester. Students were familiar with each other but did not necessarily know each other. Groups were diverse with respect to gender (Teachman index had a mean = 0.34, standard deviation = 0.28 and median = 0.5). Group collective intelligence was measured at the beginning of the 3-month period while using the task battery and procedure described in Engel et al. (2014) and Woolley et al. (2010). Participants were seated in front of computers and had to complete the group collective intelligence battery while communicating via text chat only. The completion of the battery took 62 min in total. The battery consists of a variety of tasks in line with well-developed task taxonomies (McGrath, 1984; Larson, 2010), and includes generating, choosing, remembering, executing, and sensing task types. As an example, for execution tasks, which require careful coordination of psychomotor skills of team members, team members have to jointly type and reproduce long pieces of text and numbers as accurately as possible; for sensing tasks, which require groups to recognize patterns in a noisy stimulus that is calibrated to exceed the capabilities of an individual, team members have to study a large grid of images and words and answer questions about them such as naming the most frequent object in that grid (for a detailed description of the battery see Engel et al., 2014). To calculate the collective intelligence score, we performed a factor analysis of the groups' scores on all the tasks and derived the first factor that emerged from all the groups' scores. We used this score as the measure for group collective intelligence, in line with Woolley et al. (2010) and Engel et al. (2014).

Social sensitivity was measured with Reading the Mind in the Eyes test (Baron-Cohen et al., 2001). The test consists of 36 images with eye-regions of the face of various individuals, each representing a particular mental state (e.g., arrogant, desire, insisting). Participants had to choose among four options the mental state that was represented in the image. The correct scores were summed up, with 36 being the possible maximum score for the task. In a first step, participants were asked to complete the task individually without talking to each other. In each group the members with the highest and the lowest score for the task were identified and were further used in the analyses as representing the maximum social sensitivity score and the minimum social sensitivity score. In a second step, participants were asked to come together as a group, discuss the 36 images and decide together which option was associated with the mental state represented in each of the images. Each group generated one set of solutions for the task and this score was further used in the analyses as the *group* social sensitivity score.

### Ethics statement

According to the Dutch national ethical guidelines, studies aimed at developing or evaluating professional tests and studies based on questionnaires that do not require any personal data with the potential to embarrass the participants are exempt from ethical committee approval. Because one of the aims of our research was to evaluate the CI battery in the Dutch context and our research was carried out during regular curricular activities, we did not ask for further approval from the local IRB.

## Results

All four indicators of social sensitivity (minimum, maximum, mean group score and *group* social sensitivity score) are positively and significantly correlated with the CI factor. First, the maximum and the minimum score of social sensitivity are positively and moderately associated with CI, with *r*_(30)_ = 0.41, *p* < 0.05 and respectively *r*_(30)_ = 0.52, *p* < 0.01 (Table 1). This indicates that the higher the maximum and the minimum individual scores of social sensitivity in the group are, the higher the level of collective intelligence is. To be noted, the minimum score has the strongest correlation with CI among all four predictors. In line with the results reported in Woolley et al. (2010), the mean social sensitivity is also positively associated with CI, *r*_(30)_ = 0.48, *p* < 0.01, i.e., the higher the within-group average of members' social sensitivity scores is, the higher collective intelligence of the group is. Finally, the *group* social sensitivity score has the weakest positive and significant association with CI, *r*_(30)_ = 0.38, *p* < 0.05. This indicates that the emergent, group-level social sensitivity has weaker positive association with the CI. Moreover, all four social sensitivity indicators positively correlate with each other. The strongest associations are between mean social sensitivity and maximum social sensitivity score, *r*_(30)_ = 0.81, *p* < 0.01, and between mean social sensitivity and minimum social sensitivity score, *r*_(30)_ = 0.82, *p* < 0.01. This is expected, given that the mean social sensitivity score includes both the maximum and the minimum social sensitivity score in the group.

**Table 1:** **Descriptive statistics and correlations**.

**Variables**	***Mean***	***SD***	**1**	**2**	**3**	**4**
1. CI	0	1				
2. Min social sensitivity	19.51	2.81	0.52^***^			
3. Max social sensitivity	25.29	2.06	0.41^**^	0.46^***^		
4. Mean social sensitivity	22.84	1.84	0.48^***^	0.82^***^	0.81^***^	
4. Group social sensitivity	29.25	2.60	0.38^**^	0.51^***^	0.73^***^	0.75^***^

*N = 30; Min Social Sensitivity = the lowest score in the group in the individual version of social sensitivity task; Max Social Sensitivity = the highest score in the group in the individual version of social sensitivity task; Mean Social Sensitivity = the average of group members' scores in the individual version of social sensitivity task; Group Social Sensitivity = the unique score of the group in the group version of social sensitivity task*.

** *p < 0.05*,

*** *p < 0.01*.

As shown in Table 1, the average *group* social sensitivity is 29.25, while the average maximum social sensitivity score equals 25.29, and the average mean social sensitivity (across all groups) is 22.84. This pattern of results shows that the emergent, group level social sensitivity is higher than the individual social sensitivity.

In order to test our hypotheses we ran an OLS regression analysis with minimum and maximum score of social sensitivity in the group as well as *group* sensitivity score as predictors. In order to avoid multicollinearity issues all variables have been centered to their mean. We controlled for group size given that group size varied in our sample. The results indicate that the minimum social sensitivity score is the only significant and positive predictor of CI, β = 0.49, *p* < 0.05 (Table 2, Model 2). The maximum and *group* social sensitivity scores do not predict collective intelligence with β = 0.19, *p* > 0.05 and β = −0.07, *p* > 0.05 respectively. Group size is not significantly associated with CI, with β = 0.25, *p* > 0.05. The overall model is significant with *F* = 3.72, *p* < 0.01 and it explains 27% of the variability of collective intelligence, with Adjusted *R*^2^ = 0.27. In order to check the robustness of our findings we ran the analyses without the control variables and the positive relationship between the minimum social sensitivity score and CI remained significant, β = 0.41, *p* < 0.05. The other social sensitivity predictors remained non-significant just like in the previous model. The overall model is significant with *F* = 4.07, *p* < 0.05 and it explains 24% of the variability of collective intelligence, with Adjusted *R*^2^ = 0.24 (Table 2, Model 1). The lack of differences between the two models indicates that our results are robust.

**Table 2:** **Results of regression analysis of social sensitivity on CI**.

**Independent variables**	**Model 1 β**	**Model 2 β**
Group size		0.25
Min social sensitivity	0.41^**^	0.49^**^
Max social sensitivity	0.19	0.19
Group social sensitivity	0.04	−0.07
*F*-value	4.07^**^	3.72^**^
Adj R^2^	0.24	0.27

** *p < 0.05*.

Given that there is a high correlation between the mean sensitivity, maximum sensitivity score and minimum sensitivity score, we could not introduce all the variables in one regression analysis due to multicolinearity concerns. In order to disentangle the influence of the minimum score on top of the mean social sensitivity score, we carried out an additional set of analyses. We computed an additional variable: Mean social sensitivity1 = (Sum of sensitivity scores in the group − minimum social sensitivity score in the group)/(Group size−1). This new variable represents the mean group score on social sensitivity without the lowest individual score in the group. First, we tested the influence of Mean social sensitivity1 on collective intelligence, with and without group size as a control (Model 1 and 2, Table 3). The results indicate that Mean social sensitivity1 is not a significant predictor of collective intelligence, with β = −0.07, *p* > 0.05 and respectively β = 0.01, *p* > 0.05 (while controlling for group size). Second, we tested a model in which we included all the sensitivity indicators (VIF < 3), with and without group size as a control (Model 3 and 4, Table 3). The only significant predictor is the minimum social sensitivity score, with β = 0.48, *p* < 0.05 and β = 0.44, *p* < 0.05 (while controlling for group size). These results provide additional support for the lowest social sensitivity score being the strongest predictor of CI in the group.

**Table 3:** **Results of additional regression analysis of social sensitivity on CI**.

**Independent variables**	**Model 1 β**	**Model 2 β**	**Model 3 β**	**Model 4 β**
Group size		0.23	0.16	
Mean social sensitivity1	−0.07	0.01	−0.20	−0.27
Min social sensitivity			0.48^**^	0.44^**^
Max social sensitivity			0.30	0.34
Group social sensitivity			−0.08	−0.03
*F*-value	0.15	0.73	3.21^**^	3.88^**^
Adj R^2^	−0.03	−0.01	0.27	0.28

** *p < 0.05*,

*Mean social sensitivity1 = the average social sensitivity score in the group, excluding the lowest individual social sensitivity*.

## Discussion

Our results are consistent with research on the role of group members' personality on group dynamics and effectiveness that showed that the predictive role of the lowest individual member score is superior to the predictive role of the maximum individual member score (Barrick et al., 1998; Van Vianen and De Dreu, 2001). Barrick et al. (1998) as well as Van Vianen and De Dreu (2001) found that the lowest individual score for agreeableness, extraversion and conscientiousness significantly predicted group performance. In a similar vein, we show that the score of least socially sensitive group member is predictive of collective intelligence. In other words, highly collectively intelligent groups are those in which the least socially sensitive group member has a rather high score on social sensitivity or in groups where members are not socially sensitive collective intelligence is less likely to emerge. The collective intelligence battery consists of tasks that mostly require a particular level of interdependence among the group members. When one of the group members is not able to “tune in” with the rest of the group, this might create coordination losses (Steiner, 1972) and eventually hamper the progress of the task. This is also in line with the study by Prewett et al. (2009) that shows that the minimum score in personality traits is particularly relevant for tasks with intensive workflow patterns, where work unfolds freely and frequently among all team members. Such workflow patterns require intense coordination, and while one highly sensitive member cannot compensate for the rest of the group, one highly insensitive member can disrupt the flow of coordination and diminish the level of collective intelligence of the group.

In line with our predictions, group social sensitivity is positively associated with CI, yet after introducing the minimum social sensitivity score in the regression, this positive association turned out to be not significant. Therefore, the strongest predictor for CI is the lowest social sensitivity score in the group. Since our study focuses only on the predictive power of the compositional and compilational aspects of social sensitivity, future studies could focus on further detailing the mechanisms at play.

An interesting result that emerged is that groups, when working together, scored significantly higher on the social sensitivity task as compared to aggregates of individual social sensitivity, as seen in maximum and mean individual scores. The group synergy stream of research explores the extent to which groups perform better than individuals in various types of tasks (Larson, 2010); these results indicate that the combination of individual (social) judgments during social interactions generates group synergy. Previous empirical results show that collective performance in cognitive judgmental (estimations with correct answers that are difficult to demonstrate) and cognitive decision-making (deciding among a limited number of options) tasks rarely exceeds the performance of the best individual in the group (Curşeu et al., 2013; Meslec and Curşeu, 2013). The task used in the current study could also be considered a decision-making task given that the group members had to choose one option among four alternatives for each pair of eyes. Nevertheless, the content of the task is rather different, as it refers to social judgments. The results reported in this study suggest that the content of the task may influence the extent to which groups manage to achieve strong synergy or the extent to which they manage to become better than the best member in their group. The finding that social interactions within groups are conducive for strong synergy in tasks requiring social judgments (the *group* social sensitivity exceeds the maximum social sensitivity score within the group), opens new venues for research in group synergy. Future research could explore, using within-group designs, whether the synergetic potential of groups is domain specific (the group can achieve cognitive synergy in a single type of task) or general (the group achieves synergy in several types of tasks).

Our study has several theoretical and practical implications. First, we replicate the findings of Woolley et al. (2010) in a different cultural setting and we replicate that social sensitivity (as the mean score of individual member social sensitivity) is correlated to the collective intelligence of the groups. Further, as the mean social sensitivity is a composite score that includes both the minimum and the maximum score in the group, we argue that it can hide potential effects of these two scores on collective intelligence, and show that the significant correlation of the mean sensitivity score with collective intelligence is driven by the minimum social sensitivity score in the group. Second, we refine these findings further by trying to disentangle the compositional and compilational factors that explain the positive association between social sensitivity and collective intelligence. In general, we find that group members cannot compensate for the lack of social sensitivity of other group members. Harmonious interpersonal interactions in groups, likely to be conducive for collective intelligence, require that even the least socially sensitive member scores relatively high on social sensitivity. In addition to theoretical implications, this research has implications for group design as well since it indicates that for a group to be able to consistently perform well on a wide range of tasks it needs to be composed of members that are highly socially sensitive.

## Author contributions

NM, PLC, IA: contributed to the design of the work; NM, IA: contributed to the acquisition of data; PLC, NM, IA: contributed to the analysis and interpretation of data; PLC, NM, IA: contributed to the drafting and revising of the work; MN, PLC, IA: approved the final version of the work.

## Funding

PLC was supported by a grant of the Romanian National Authority for Scientific Research, CNCS – UEFISCDI, project number PN-II-ID-PCE-2011-3-0482. The funders had no role in study design, data collection and analysis, decision to publish, or preparation of the manuscript.

### Conflict of interest statement

The authors declare that the research was conducted in the absence of any commercial or financial relationships that could be construed as a potential conflict of interest.
